# Spread of *Toxoplasma gondii* among animals and humans in Northern Italy: A retrospective analysis in a One-Health framework

**DOI:** 10.1016/j.fawpar.2023.e00197

**Published:** 2023-06-08

**Authors:** F.M. Dini, S. Morselli, A. Marangoni, R. Taddei, G. Maioli, G. Roncarati, A. Balboni, F. Dondi, F. Lunetta, R. Galuppi

**Affiliations:** a Department of Veterinary Medical Sciences, University of Bologna, Italy; b Department of Medical and Surgical Sciences, University of Bologna, Italy; c Istituto Zooprofilattico Sperimentale Della Lombardia e della Emilia-Romagna, Italy; d Microbiology Unit, IRCCS Azienda Ospedaliero-Universitaria di Bologna, 40138 Bologna, Italy

**Keywords:** Toxoplasmosis, Epidemiology, Humans, Animals, Emilia-Romagna region, Serology

## Abstract

Toxoplasmosis occurs worldwide and is considered one of the most important food-borne parasitic zoonoses. The consumption of undercooked meat containing viable tissue cysts and ingestion of environmental oocyst are the most important sources of infection. The aim of this retrospective study was to evaluate the spread of *Toxoplasma gondii* in the province of Bologna (Emilia-Romagna region) in northern Italy, with a One Health approach, comparing seropositivity rates in different animal species and in humans over the last 19 and 4 years respectively. Analyses were performed on serological data collected over different periods at three separate locations: Istituto Zooprofilattico Sperimentale della Lombardia e della Emilia-Romagna (IZSLER); Veterinary University Hospital Clinical Pathology Service, Department of Veterinary Medical Sciences, University of Bologna; and Unit of Microbiology, St. Orsola Hospital, Bologna. Most relevant seropositivity rates observed in animals were 15.5% (wild boar), 25% (roe deer), 18.7% (goat), 29.9% (sheep), 9.7% (pigs), 42.9% and 21.8% in cat and dog, respectively. A comprehensive screening was conducted on a population of 36,814 individuals, revealing a prevalence of 20.4%. Among pregnant women, a frequence of 0.39% for active toxoplasmosis was observed. Despite certain limitations, this study provided valuable insights into the extensive distribution of this parasitic infection among diverse animal species and human populations in the province of Bologna. These findings underscore the importance of implementing consistent and proactive toxoplasmosis screening protocols during pregnancy, while emphasizing the critical need for adopting a One Health approach for effective control of this parasitic disease.

## Highlights

• T. gondii serological data from animals and humans were retrospectively analysed.
• Seropositive wild and domestic animals are uniformly distributed in the territory.
• In humans 161 (0.44%) *T. gondii* active infection were observed in a 4 year period.
• Multidisciplinary approach is crucial in the control of Toxoplasmosis.

## 1 Introduction

Toxoplasmosis is a zoonotic parasitic infection with a worldwide distribution caused by the apicomplexan protozoon *Toxoplasma gondii*. The sexual reproduction of the parasite occurs in felids, definitive hosts, that play an essential role in the contamination of the environment with oocysts, whereas a broad range of warm-blooded animals, including humans, act as intermediate hosts (Dubey et al., 2020a). Toxoplasmosis is recognized as one of the most significant food-borne parasitic zoonoses worldwide, and it is estimated that approximately one third of the global population is infected with *T. gondii*. (Montoya and Liesenfeld, 2004) In immunocompetent individuals, *Toxoplasma* infection is typically benign and self-limiting. However, it can cause life-threatening disease in fetuses and immunosuppressed individuals. Recent studies suggest that latent *Toxoplasma* infection may be associated with the development of specific neuropsychiatric conditions (Tyebji et al., 2019). The primary sources of infection are the consumption of undercooked meat containing viable tissue cysts and the ingestion of food and water contaminated with oocysts (Pereira et al., 2010, Guo et al., 2015). The development of a serological assay capable of differentiating oocyst-induced infection from bradyzoite-induced infection has facilitated the recognition of oocysts as the primary reservoir of infection during outbreaks in the United States (Hill et al., 2011; Boyer et al., 2011). Nonetheless, the widespread implementation of these diagnostically valuable epidemiological techniques remains limited, underscoring the necessity for a meticulous transdisciplinary approach to effectively prevent and control this parasite (Djurkovi'c-Djakovi'c et al., 2019).

In Southern Italy, a recent monitoring program has been implemented with the aim of enhancing the epidemiological knowledge regarding toxoplasmosis and identifying the risk factors associated with the infection in both animals and humans. This program adopts a multi-institutional approach to comprehensively investigate the subject (Pepe et al., 2021). In the Emilia-Romagna region of Italy, a three-year prospective observational study has shed light on the prevalence of toxoplasmosis among pregnant women. The study revealed that 22.3% of women tested positive for toxoplasmosis during early pregnancy. Notably, non-native women originating from Africa, Asia, Eastern Europe, and South America exhibited a higher likelihood of acquiring the infection during pregnancy compared to Italian women. Furthermore, the incidence rate of toxoplasmosis in this region was found to be higher than that reported in other European countries (Capretti et al., 2014). Serological analyses conducted during the same period in the Emilia-Romagna region also revealed a relatively high prevalence of toxoplasmosis in sheep flocks, reaching 41.9% (Parigi, 2014). Recently, a molecular investigation revealed a prevalence of 14% for parasite infections among wild water birds hunted in the aforementioned area (Dini et al., 2023).

To effectively implement appropriate control measures aimed at reducing the incidence of congenital toxoplasmosis, it is crucial to gain a comprehensive understanding of the extent of *T. gondii* circulation within the specific area of interest. Therefore, the objective of this study was to assess the prevalence of *T. gondii* infection in the province of Bologna, located within the Emilia-Romagna region of Italy, utilizing a One Health approach. The study aimed to compare the seroprevalence of *T. gondii* across various animal species with the seropositivity data observed in humans over the past years. By adopting this multidisciplinary approach, a more comprehensive and integrated understanding of the infection dynamics can be achieved, facilitating the development of targeted control strategies.

## 2 Materials and methods

The data utilized in this study were obtained through a retrospective analysis of serological investigations conducted at two distinct veterinary institutions and one human hospital.

Specifically, information pertaining to animal infections was extracted from the databases of the Istituto Zooprofilattico Sperimentale della Lombardia e della Emilia-Romagna (IZSLER) covering the period from 2002 to 2021. Similarly, data from the Veterinary University Hospital (VUH), specifically the Clinical Pathology Service of the Department of Veterinary Medical Sciences at the University of Bologna, were collected for the period from 2006 to 2021. This involved retrieving information from all samples that underwent serological testing for *T. gondii*.

IZLER is an Italian public health institute that is engaged in control and research initiatives, as well as offering services in the domains of animal health, food safety, and zoonoses. Within their scope of activities, IZLER conducts serological tests on diverse domestic and wild animal species for both routine institutional screening and diagnostic purposes upon request. During the specified period, various tests were employed at IZSLER: Latex Agglutination Test (LAT, Toxotest; Eiken Chemical, Tokyo, Japan), Enzyme-Linked Immunosorbent Assay (ELISA, ID Screen® Toxoplasmosis Indirect Multi-species; ID-Vet - Innovative Diagnostics, Grabels, France), and Immunofluorescence Antibody Test (IFAT, Toxo-Spot IF; bioMérieux, Marcy-l'Étoile, France). IFAT was performed using a commercial antigen (Toxo-Spot IF; bioMérieux) and, as conjugate, Anti-Dog and Anti- Cat IgG of IZSLER internal production were used. Antibody titer ≥1:40 was considered positive for IFAT, while antibody titer ≥1:32 was considered positive for LAT, as suggested by the manufacturers.

At the VUH, dogs and cats were tested for diagnostic purpose only by the means of IFAT (MegaFLUO TOXOPLASMA g, MegaCor Diagnostik, Hoerbranz, Austria) using Anti-Dog IgG-FITC antibody (Sigma-Aldrich, Saint Louis, MO, USA) and FITC IgG conjugate Anti-Cat (MegaCor Diagnostik, Hoerbranz, Austria); antibody titer ≥1:40 was considered positive.

In both laboratories, in addition to the detection of specific IgG antibodies, IFAT was also used for the detection of specific IgM antibodies. Concerning humans, this study encompassed all individuals who underwent immune status evaluation for *T. gondii* infection at the Unit of Microbiology in St. Orsola Hospital. This particular hospital serves the entire population in the metropolitan city of Bologna and its province, which consists of over 1 million inhabitants. The hospital's microbiology laboratory, equipped with an online database called DNLAB® (Dedalus), contains records of all tests conducted in the past four years (2018–2021). For serological analysis of human serum samples chemiluminescence immunoassays (Elecsys Toxo IgG and Elecsys Toxo IgM, Roche Diagnostics GmbH, Mannheim, Germany) were initially employed to detect IgM and IgG antibodies. Borderline or positive IgM screening results were subsequently confirmed using enzyme-linked fluorescent assays (ELFAs) (Vidas Toxo IgM, bioMerieux, Marcy l'Etoile, France), to exclude IgM residual. If IgM positivity was confirmed, and IgG Avidity test (Vidas Toxo IgG Avidity, bioMerieux) was performed. Positivity in ELFAs and low Avidity Index were indicative of *T. gondii* active infection.

Data collected from animals were organized in databases. The two laboratories, IZSLER and VUH, provided different variables. For IZSLER, information included the animal species examined, the municipality of origin (Bologna province consists of 55 municipalities), and the date of sample submission. On the other hand, VUH provided additional data such as age, sex, breed, municipality of origin (based on owner address), the date of testing, and associated clinical information. All possible duplicate observations were removed with the first occurrence of the animal in the dataset retained.

Pearson's χ2 test was used to associate species and (when available) age, sex, and, for the cat, the origin (owned or unowned) with seroprevalence data. The level of statistical significance was 5% (*P* < 0.05).

For humans, data collected included prevalence and the number of active infections (positive IgM ELFA analyses and low avidity samples).

The Sample Size Calculator (https://www.surveysystem.com/sscalc.htm) was used to calculate 95% confidence intervals (CIs) for the observed prevalence values.

## 3 Results

Between 2002 and 2021, the Istituto Zooprofilattico Sperimentale della Lombardia e dell'Emilia Romagna (IZSLER) conducted serological testing for *Toxoplasma gondii* infection on a total of 4263 sera derived from various animals, including those from farms, companion animals, and wild species (Table 1). Specifically, investigations were carried out on small ruminants, such as sheep and goats, in response to abortion outbreaks. Among the 64 goat samples examined, 12 samples (18.7%) tested positive. Notably, in the municipality of San Giovanni in Persiceto, clusters of positive cases were identified in three different years (2003, 2004, 2019). Out of the 117 sheep tested, 35 (29.9%) were found to be positive. Additionally, as part of a food safety research project, 31 pigs from the same municipality in the Bolognese Apennines were examined, and the observed frequency of positive cases was 9.7%. Concerning wild animals, of a total of 594 wild boar and 104 roe deer examined, 15.5% and 25% were seropositive, respectively, indicating infection was significantly more frequent in roe deer that in wild boar (*P* = 0.025). Very few wild carnivores were tested to allow for meaningful results, but it is noteworthy that one of the 5 wolves and both red foxes tested were seropositive. In contrast, none of the 34 tested hares was seropositive. Pigeons (*n* = 105), tested during a population control campaign, showed a seroprevalence of 3.8%. Among companion animals, the species most represented was the cat (*n* = 3087), which tested positive for total antibody (ELISA, LAT) or IgG in 1355 cases (43.9%). In most cases (3082) the sampling was carried out between 2002 and 2006. Evaluating stray cats from colonies versus owned cats, the former had a statistically higher seroprevalence (46.5% vs. 33.4%; χ^2^ = 34.14, *P* < 0.001). In dogs (*n* = 114), the frequency observed was lower (19.3%) with 22 positive dogs.

**Table 1:** animal species tested at IZSLER, number of animals tested, number of municipalities of origin, frequency of seropositivity for *T. gondii* antibodies, confidence interval, number of municipalities of origin of positive animals and serological method used.

Animal Species	n. examined animals	n. municipalities of origin	Seropositive n (%) [CI 95%]	n. municipalities of origin of positive	Test Used
Goat	64	9	12 (18.7%) [12.45–24.95]	4	ELISA, LAT
Sheep	117	14	35 (29.9%) [21.6–38.2]	11	ELISA, LAT, IFAT
Pig	31	1	3 (9.7%) [0–20.12]	1	ELISA
Rabbit	6	2	0 (0%) [n.d.]	0	LAT
Roe Deer (*Capreolus capreolus*)	104	25	26 (25%) [16.68–33.32]	18	ELISA
Wild Boar (*Sus scrofa*)	594	19	92 (15.5%) [12.59–18.41]	14	ELISA
Hare (*Lepus europaeus*)	34	2	0 (0%) [n.d.]	0	IFAT
Pidgeon (*Columba livia*)	105	5	4 (3.8%) [0.14–7.46]	2	LAT
Wolf (*Canis lupus*)	5	5	1 (20%) [0–55.05]	1	ELISA
Red Fox (*Vulpes vulpes*)	2	2	2 (100%) [n.d.]	2	ELISA
Dog	114	21	22 (19.3%) [11.9–26.54]	11	ELISA, LAT, IFAT
Cat	3087	39	1355 (43.9%) [42.89–44.91]	30	ELISA, LAT, IFAT

Concerning the results obtained from the VUH, data derived from 65 dogs and 208 cats of the province of Bologna: these were owned animals in which toxoplasmosis was a differential diagnosis based on their clinical and pathological findings. Out of 208 cats tested, 59 (28.4%) [C.I. 95%: 22.27–34.53] were positive. No significant difference has been detected in frequency of positivity between males and females (25.5% vs. 30.9%), although entire males appeared less frequently positive than neutered ones (13.6% vs 35.2%; *P* = 0.02). The seropositivity rate was 18.2% in subjects younger than one year of age, and 30.7% in cats ≥ one year, although the difference was not significant (Table 2).

**Table 2:** Signalment and serological results for *T. gondii* in dogs and cats examined at the VUH.

Cats			Positive n. (%)		Negative n. (%)		Chi-square test -P
Examined	208 [from 36 municipalities]		59 (28.4%) [from 23 municipalities]		149 (71.6%)		
Age (207 known)	< 1 year		8 (18.2%)		36 (81.8%)		NS
	≥ 1 year		50 (30.7%)		113 (69.3%)		
Sex	male	neutered	25 (25.5%)	19 (35.1%)	73 (74.5%)	35 (35.7%)	NS
		entire		6 (13.6%)		38 (38.7%)	4.84 P < 0.05
	female	neutered	34 (30.9%)	23 (30.3%)	76 (69.1%)	53 (69.7%)	NS
		entire		11 (32.4%)		23 (67.6%)	NS
Dogs							
examined	65 [from 25 municipalities]		17 (26.2%) [from 11 municipalities]		48 (73.8%)		
Age	< 1 year		1 (11.1%)		8 (88.9%)		NS
	≥ 1 year		16 (28.6%)		40 (71.4%)		
Sex	male	neutered	7 (28%)	0 (0%)	18 (72%)	2 (100%)	NS
		entire		7 (30.4%)		16 (69.6%)	NS
	female	neutered	10 (25%)	5 (35.7%)	30 (45%)	9 (64.3%)	NS
		unneutered		5 (19.2%)		21 (80.8%)	NS

Concerning dogs, the seropositivity rate observed was 26.2% (17/65) [C.I. 95%: 15.51–36.89]. There was no difference in prevalence according to sex (male or female, entire or neutered) or age (Table 2).

**Fig. 1:**
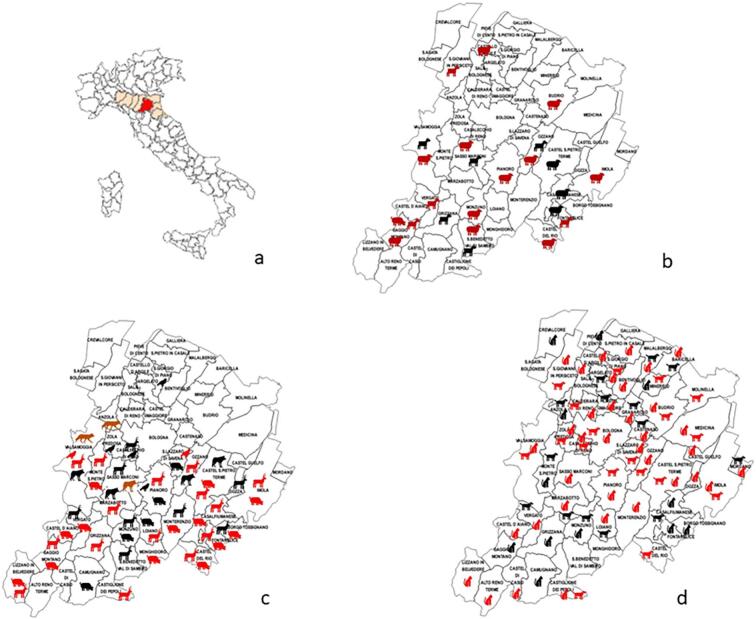
Distribution of the animals examined by IZSLER and VUH: a) province of Bologna in Emilia Romagna region (Italy); b) Livestock species; c) Wild animals; d) dog and cat. The red figure means that at least one subject tested positive. (For interpretation of the references to colour in this figure legend, the reader is referred to the web version of this article.)

Fig. 1 illustrates the distribution of animals examined by IZSLER and VUH across various municipalities in the province of Bologna. The examination of wild species predominantly occurred in the southernmost region of the province, specifically in the Apennine area. On the other hand, dogs and cats were sampled from almost all municipalities throughout the entire province. The positive animals, indicating the presence of the infection, were found to be uniformly distributed across the different areas.

In the study period of four years, a comprehensive analysis was conducted on 122,377 serum samples at the Microbiology Unit of Sant'Orsola Hospital (Table 3). It should be noted that some individuals underwent multiple testing, resulting in a total of 36,814 patients (primarily adults) being evaluated (Table 3). Among these patients, the majority were women (88.2%), with an average of 3.61 samples assessed per woman or 1.16 per man. Notably, a significant proportion of women (87.9%) underwent testing during their pregnancy.

**Table 3:** Data concerning tests and serological results for *T. gondii* in humans. In the “acute infection” category, only active *T. gondii* infection cases, with positive IgM ELFA analyses and low-avidity samples, have been grouped.

	**Total number**	**men** **(%)**	**women** **(%)**	**pregnant women** **(%)**	**non-pregnant women** **(%)**
**Screened subjects**	36,814	4341 (11.8%)	32,473 (88.2%)	28,572 (87.9%)	3901 (12.1%)
**Acute infection** **(frequence)**	161 (0.44%)	26 (0.60%)	135 (0.42%)	113 (0.39%)	22 (0.56%)

The observed prevalence of toxoplasmosis was 20.4% (95% CI: 20.8–20.0), ranging between 20.0% and 20.8% throughout the study period. There were no significant differences in prevalence based on year, season, or sex. During 2020, the number of tests conducted was markedly lower compared to other years, with a reduction of over 15%, which can likely be attributed to the impact of the COVID-19 pandemic.

IgM positivity confirmation by ELFA tests were performed on a total of 1915 sera. Among these, 504 sera (26.3%) tested positive for IgM. A diagnosis of acute *T. gondii* infection was established in 161 patients, determined by excluding cases of persistent IgM positivity and considering a low Avidity Index. This resulted in a frequency of 0.44% among the screened population. Most of the acute infection (113/161) were diagnosed in pregnant women. The frequencies of toxoplasmosis did not differ between men and women, or between pregnant and nonpregnant women.

Moreover, we divided the pregnant women into two groups: subjects attending the Family Advisory Health Centers of Bologna and its province for prenatal care and maternal screening and subjects attending the Maternal-Fetal Medicine Unit of Sant'Orsola Hospital, Bologna, for a second level toxoplasmosis diagnosis (women with clinical, echographic or laboratory suspicion of toxoplasmosis). In particular, in the former case, the frequence was 0.25% (69/27,513 subjects) and in the latter the value was 4.1% (44/1059), with *p* *=* 0.0001.

## 4 Discussion

In this study, a One Health approach was employed to analyse data concerning the presence of *Toxoplasma gondii* in the province of Bologna. The analysis focused on the key host species that play a significant role in the epidemiology of the parasite.

While the National Health Service has facilitated the collection of data on human health for the province through centralized analyses, the veterinary field presented a fragmented scenario due to the involvement of numerous private laboratories primarily focused on domestic animals and lacking a shared database. To overcome this limitation, data was obtained from two major institutional veterinary centres involved in diagnostic activities within the territory, resulting in a substantial number of samples from various animal species collected over the past 20 years. It is important to acknowledge that this approach has certain limitations. Firstly, the sampling methods varied across different categories, with some samples obtained through diagnostic processes and others through regional surveillance plans for toxoplasmosis in animals. Consequently, certain animal species may be underrepresented in terms of the number of specimens. Additionally, the available databases cover different time periods (2002 to 2021 for IZSLER, 2006 to 2021 for VUH, and 2018 to 2021 for St. Orsola Hospital), and the serological tests employed differ not only between different diagnostic centers but also within the same diagnostic unit at different time points. Nonetheless, despite these limitations, the collected data provides an overall understanding of the circulation of *T. gondii* in various animal categories and humans in the province of Bologna.

### 4.1 Livestock

In this retrospective study, diagnostic confirmation for *Toxoplasma gondii* was conducted in 181 small ruminants in the province of Bologna, specifically following abortion outbreaks, with seroprevalences of 29.9% in sheep and 18.7% in goats. The significance of toxoplasmosis in these animal species extends to both public health and economic aspects. They are considered a primary source of infection among certain ethnic groups that consume undercooked meat due to cultural reasons (Kijlstra and Jongert, 2008). Furthermore, *T. gondii* is recognized as one of the primary causes of abortion in sheep and goat farming (Dubey, 2022). Such losses can be particularly devastating, especially for small family farms, which are prevalent in Mediterranean regions and have reported abortion rates as high as 75% (Edwards and Dubey, 2013). Although the seroprevalence values observed in our study were comparatively lower than those reported in other studies conducted in Northern Italy (Parigi, 2014, Gazzonis et al., 2015, Gazzonis et al., 2020), it is crucial to consider the specific context of our data collection. The samples we analysed were obtained from farms in the area after abortion outbreaks, and it is possible that these outbreaks were not solely attributed to *T. gondii* infection. Previous studies have shown that goats generally exhibit lower positivity rates compared to sheep, consistent with the findings of the present survey. This variation can be attributed to the distinct feeding behaviors of the two species. Sheep, as grazers, are more vulnerable to the exposure of *T. gondii* oocysts and other soil-borne parasites, as indicated by prior research (Hoste et al., 2010). In contrast, goats, being browsers, have a relatively lower risk of contracting this parasitic infection.

### 4.2 Wild animals

The observed seroprevalences in wild boar (15.5%) and roe deer (25%), do not seem to reflect the increasing of the prevalence throughout the trophic chain previous described (Smith and Frenkel, 1995; Ferroglio et al., 2014) and are particularly intriguing: the higher exposure found in herbivores, suggests a relevant role of oocysts contamination in the considered territory. Oocyst environmental contamination could be linked to the presence of wild and domestic felids (Otranto et al., 2015). In recent years, the populations of wild boars and roe deer in Europe have shown significant expansion despite being among the most heavily hunted ungulate species (European Food Safety Authority (EFSA), 2014; Milner et al., 2006; Pittiglio et al., 2018). In recent decades, the roe deer population has undergone significant migration from northeastern regions to northwestern areas and the Apennines, establishing a relatively stable presence in our territories, especially in proximity to human-altered areas with a high abundance of free-roaming cats (Carnevali et al., 2009). The consumption of wild ungulate meat poses a potential risk of infection for other carnivorous hosts, including humans (Tenter et al., 2000). The meat of these ungulates is highly valued in certain regions of Italy, where culinary traditions may include the preparation of raw dishes.

Despite the limited sample size of wild carnivores in this survey, a notably high seroprevalence was observed, with 2 out of 2 red foxes and 1 out of 5 wolves testing positive for *T. gondii* antibodies. These findings are consistent with the results obtained in other surveys, where seroprevalence rates of up to 84.7% were reported in red foxes from north-eastern Europe (Kornacka-Strackonis, 2022), and *T. gondii* DNA was detected in 20% of grey wolves in Serbia (Uzelac et al., 2019). The higher susceptibility of these hosts to *T. gondii* infection, resulting from the consumption of both tissue cysts and environmentally-transmitted oocysts, positions them as valuable sentinels for monitoring the presence of the parasite within specific territories.

### 4.3 Dog and cat

Overall, in this study, cats demonstrated a seroprevalence of 42.9%, (1414 out of 3295 tested cats from both laboratories), in line with the estimate prevalence in Europe (43%) (Montazeri et al., 2020). Notably, colony cats exhibited significantly higher seroprevalence (46.5%) compared to owned cats (32.1%) (*P* < 0.001). Studies conducted in central Italy reported similar prevalence, such as 44% in colony cats from Florence province (Mancianti et al., 2010), and 62% in Rome (Macrì et al., 2009). However, it is important to consider the limitations of comparing these data due to variations in diagnostic techniques used over time, including in our laboratories, and the different cutoff titers.

Across the province of Bologna, seropositive cats were discovered in 36 out of 55 municipalities, indicating a widespread distribution of infected cats throughout the territory. This includes hilly and mountainous areas of the province, where positive cases were also found among wild ungulates. The detection of specific anti-*T. gondii* IgG in cats holds significant epidemiological implications. Cats that have developed protective IgG antibodies against *T. gondii* have likely shed oocysts in their living environment at some point, either in the distant or recent past, but are generally considered immune to further shedding of the parasite (Dubey, 1995).

Although not epidemiologically comparable to cats, dogs' exposure to *Toxoplasma* also has public health implications. Dogs can act as mechanical carriers of *T. gondii* oocysts, excreting them in their feces after ingestion from cat stool. Moreover, *T. gondii* oocysts can contaminate dog fur, potentially leading to human infection through contact with the dog's coat, mouth, and feet (Lindsay et al., 1997; Dubey et al., 2020b).

### 4.4 Humans

The seropositivity rate (20.4%) observed in this study is similar to the ones previously reported in other areas of Italy (Mosti et al., 2013; Fanigliulo et al., 2020).

The majority of available information on *Toxoplasma* seroprevalence in humans primarily focuses on women of reproductive age, utilizing prenatal screening data. Even in our retrospective study, over 95% of serological tests for *T. gondii* conducted between 2018 and 2021 were carried out on samples obtained from females, while only 4.1% were from males. This finding is not surprising, as serological testing during early pregnancy is strongly recommended (although not mandatory) according to the guidelines of the National Public Health Service. Moreover, *T. gondii* serology is provided free of charge to all pregnant women by the Italian Government (Italian Government, 2017). Consequently, pregnant women undergo testing for *T. gondii* during early pregnancy, and seronegative women are advised to undergo subsequent testing every 4–6 weeks until delivery. In addition to pregnant women, our study also included screenings conducted on transplant patients and individuals with clinical suspicion of toxoplasmosis.

In various high-income countries, a decline in *T. gondii* seroprevalence among the human population, specifically women of childbearing age, has been observed since 2001 (Milne et al., 2023). This decrease has been attributed to shifts in dietary patterns, reduced prevalence in intensively farmed livestock, improved hygiene practices, and enhanced health education, collectively resulting in decreased exposure to the parasite (Pinto et al., 2012; Martini et al., 2020; Milne et al., 2023).

Although in many contexts, declining exposure to *T. gondii* commonly lead to a lower incidence of congenital toxoplasmosis due to fewer seroconversions in pregnancy, some studies have observed an unexpected increase in congenital toxoplasmosis incidence or IgM prevalence despite declining seroprevalence (Edelhofer and Prossinger, 2010; Mongua-Rodriguez et al., 2013). This phenomenon aligns with the epidemiological concept of “peak shift” dynamics, which suggests that as infection rates decrease, the risk of exposure is shifted to a higher age group (Woolhouse, 1998). Consequently, a naive population, including women of childbearing age, may become more susceptible to the infection due to increased exposure.

In the screened pregnant women population, the frequency of acute infections decreased to 0.25% (from the 0.39% of acute infection rate in the total population), which is consistent with findings from a previous study involving pregnant women in the Emilia-Romagna region (Billi et al., 2016). However, in the “second level *T. gondii* diagnosis group,” the incidence was notably higher at 4.1%. Another study (Capretti et al., 2014) previously reported a significantly elevated incidence of toxoplasmosis among pregnant women who were non-native. A major limitation of our current survey is the inability to distinguish between native and non-native women, as well as the lack of clinical information and specific details regarding dietary habits.

Considering the seroprevalence in various animal species and humans observed in our specific study area, prenatal screening programs remain the mainstay of the prevention of congenital toxoplasmosis, allowing the early identification of maternal infection cases. An early detection of infection permits to start promptly the antenatal treatment to interrupt the vertical transmission, underlining the need to maintain an appropriate and active screening for toxoplasmosis during pregnancy.

## 5 Conclusions

The findings of this study provide compelling evidence for the wide distribution of the parasite in the study area. Specific antibodies were detected in a range of wildlife, livestock, domestic animals, and humans, indicating a constant presence of the parasite in diverse environments. The seropositivity rates observed in wildlife species like roe deer and wild boars underscore their significance in the parasite's epidemiology. They serve as indicators of environmental contamination in both wild and peri-urban settings, as well as potential sources of infection for humans. Furthermore, the presence of seropositivity in human populations, as well as in domestic and companion animals, highlights the occurrence of the parasite in anthropized environments. The interplay between anthropogenic and environmental factors shapes the epidemiology of this parasitic infection and influences its spread. Given the interdisciplinary nature of this issue, a One Health approach is crucial not only in prevalence surveys like this study but also in control, education, and prevention campaigns.

## Declaration of Competing Interest

The authors declare that they have no known competing financial interests or personal relationships that could have appeared to influence the work reported in this paper.
